# High-potency PD-1/PD-L1 degradation induced by Peptide-PROTAC in human cancer cells

**DOI:** 10.1038/s41419-022-05375-7

**Published:** 2022-11-04

**Authors:** Meng-Yuan Dai, Yu-Ying Shi, An-Jin Wang, Xue-Lian Liu, Miao Liu, Hong-Bing Cai

**Affiliations:** 1 Department of Gynecological Oncology, Zhongnan Hospital of Wuhan University, Wuhan, China; 2 Hubei Key Laboratory of Tumor Biological Behaviors, Wuhan, China; 3 Hubei Cancer Clinical Study Center, Wuhan, China; 4 Department of Pathology, Brigham and Women’s Hospital, Harvard Medical School, Boston, MA USA

**Keywords:** Tumour immunology, Drug development

Immune checkpoint molecules are important in differentiating between “friend and foe” in the human immune response and are particularly well known for their impact on anti-cancer immunity. The checkpoint protein programmed cell death protein 1 (PD-1), in particular, plays a key role in suppressing the T cell-mediated immune response. The interaction of PD-1 with its main ligand, PD-L1, activates signaling pathways which inhibit T cell function, which is normally important to avoid excessive immune activation and autoimmunity [1]. However, high PD-L1 expression in tumor cells can allow them to escape the immune system. Blocking the PD-1/PD-L1 pathway is considered a promising approach to improve cancer immunotherapy, but so far, the successful use of PD-1/PD-L1 inhibitors has been hindered by low efficiency and consistent drug-resistance [2]. Degradation technologies for proteins of interest (POIs), such as the proteolysis-targeting chimera (PROTAC) system, have huge potential for overcoming these problems [3]. However, targeted protein degradation (TPD) technologies predominantly rely on the intracellular E3 ubiquitin proteasome system (UPS) or the lysosome, a membrane-bound cell organelle that contains digestive enzymes [4]. Checkpoint proteins such as PD1 are located on the cell membrane, where they are able to bind to their ligands. Although there are a wide variety of monoclonal antibodies (mAbs) that target PD-1/PD-L1, their unsatisfactory therapeutic effects may be due to the inability of mAbs to reach to intracellular POIs [5]. Another approach is small molecular inhibitor drugs, but these require a very strong interaction with POIs in order to be effective [6]. In contrast, only a medium-low binding force is sufficient to allow PROTAC drugs to effectively target POIs for degradation due to their high-affinity binding to the E3 UPS [7]. The protein–protein interactions (PPI) between different kinds of POIs and their interacting proteins can inform the appropriate peptide-binding sites for PROTAC strategies [8, 9]. For membrane-bound POIs, it is much easier to design degradation compounds based on peptides, rather than small molecules, as the latter generally require deep folding pockets on POIs for effective binding [10]. We hypothesized that, for POIs that are difficult to target with ternary molecule PROTAC compounds, such as checkpoint proteins, peptides could be an effective alternative strategy. Here, we designed a novel system using peptides targeting PD-L1 and PD-1 connected to E3 ligase/proteasome binding moieties to induce their degradation.

Cell-intrinsic PD-1 and PD-L1 were found to be expressed in a large variety of cancer cells, with particularly high PD-1 expression in 13 tumor types and 40 tumor cell lines, including cervical cancer lines [11]. In this study, we evaluated the expression of PD-1 and PD-L1 in the two human cervical cancer cell lines C33A and Hela by western blotting (Fig. S1). We found that both proteins were highly expressed in both cervical cancer cell lines. Therefore, we used C33A and Hela as model cell lines to evaluate the degradation efficiency of our synthesized PD-1- and PD-L1-targeting Peptide-PROTACs.

The heterobifunctional peptide degraders targeting either PD-1 or PD-L1 included a Cell-Penetrating Peptide (CPP) sequence, Targeting Protein Recognition (TPR) Peptide sequence, Peptide Linker, and E3 Recruitment Peptide (ERP) sequence, labeled with a rhodamine molecule. We screened several CPP and TPR of peptides (Fig. 1a, b and Table S1) and evaluated their effect on the degradation of PD-1 and PD-L1 in C33A and Hela cells. Several Peptide-PROTACs led to successful degradation (Fig. S2), but Peptides 1 and 2 with von Hippel-Lindau (VHL) binder and CPR of “RRRRRRRR” displayed the highest degradation efficiency on PD-1 and PD-L1, respectively (Fig. 1c and S3). To design the high potency Peptide-PROTAC, we selected not only the TPR sequence based on the previous studies on efficiency blocking sites, but also optimized the peptide sequences using an artificial intelligence (AI)-directed drug design system and molecular docking. Among these peptides, the initial TPR sequence of Peptide 1 and Peptide 6 that target PD-1 and Peptide 11 that target PD-L1 were previously reported to directly bind to POIs [12–14], whereas the TPR sequence of Peptide 2 that target PD-L1 palmitoylation was obtained using AI-directed peptide design (Fig. 1b). Peptide 12 was the inhibitor sequence that target PD-L1 palmitoylation without ERP sequence. Furthermore, we tried the degradation effects of different types of CPP and ERP in other Peptide-PROTACs (Table S1). For PD-1, Peptide 1 had a higher potent degradation of target cell-intrinsic PD-1 in C33A and Hela cells compared with Peptide 6 (Fig. 1c, d), while Peptide 1 also degraded the PD-1 level of T cells and affected the T cell function by our co-culture of CD3+ T cell and cancer cells (Fig. S4). For PD-L1, we found that targeting the palmitoylation of PD-L1 was much more efficient than targeting PD-L1 directly (Fig. 1d). Inhibition of PD-L1 palmitoylation induced by the palmitoyltransferase ZDHHC3 (DHHC3) has been shown to decrease the expression of PD-L1 by attenuating its lysosomal degradation [5]. In contrast, blocking the proteasomal degradation pathway has little effect on PD-L1 degradation. First, we verified the PD-L1 palmitoylation site and demonstrated that a competitive inhibition peptide against this site decreased the PD-L1 level in cancer cells. The peptide for palmitoylation of PD-L1 was tested by parallel reaction monitoring (PRM)-based targeted mass spectrometry (Figs. S5 and S6). Our results that targeting DHHC3 via PROTAC degraded DHHC3 efficiently and importantly also led to a 16-fold higher effectiveness in PD-L1 degradation than targeting PD-L1 directly (Figs. 1d and S7). To evaluate the ability the peptides to penetrate the cell membrane, we used electronic and confocal laser microscopy. After 4 h drug incubation, our microscopy results showed that the Peptide-PROTACs with CPP sequences could successfully cross the cell membrane (Figs. 1e and S8). Our designed Peptides displayed a high degradation efficiency for PD-1 and PD-L1, but previous Peptide-PROTACs or degraders for other proteins have generally shown a relatively low degradation efficiency at a dose of 50–100 μM [8, 9], whereas we measured a significant decrease in the expression of PD-1 and PD-L1 at doses <5 μM, demonstrating the high efficiency of Peptides 1 and 2, respectively (Fig. 1d). By measuring fluorescence intensity by flow cytometry, we demonstrated the accumulation of Peptide-PROTAC in tumor cells with the increasing of dose or incubation time (Fig. 1f). The low working dose of Peptide-PROTAC required for PD-1 and PD-L1 degradation is encouraging for future translation to in vivo and clinical experiments. Moreover, we demonstrated that all of our designed Peptide-PROTACs had a low toxicity to C33A cells up to a 24 h incubation time and a dose of 50 μM, as assessed by 3-(4,5-dimethylthiazol-2-yl)-2,5-diphenyltetrazolium bromide) (MTT) assay (Fig. S9). To investigate the strength of interaction between the POIs and their peptides ligands, we monitored the induced thermal stabilization of the target protein using a cellular thermal shift assay (CETSA). This involves the generation of a melt curve, in which the induced stabilization of the target protein is monitored at a single compound concentration (25 μM) across a range of temperatures (37–61 °C) and compared with untreated samples. The results confirmed very strong interaction between PD-1/PD-L1 and their respective peptides (Fig. 1g). The efficient binding to POIs is likely to be a major reason why our designed Peptide-PROTACs display a better degradation efficiency than some previously reported Peptide-PROTACs for other POIs (Fig. 1h). In order to confirm the degradation mechanism, we blocked the proteasome pathway using MG132, a proteasome inhibitor. After incubation with 10 μM Peptide-PROTAC for 8 h, we again measured a significant decrease in PD-1/PD-L1 protein levels, but this decrease was completely suppressed by addition of MG132 in both C33A and Hela cells (Fig. 1i, j, S10, and S11). Any PD-L1 degradation induced by direct inhibition of PD-L1 palmitoylation would not be rescued by MG132, as palmitoylated PD-L1 is processed via the lysosomal pathway. The fact that the decrease in PD-L1 expression can be rescued by MG132 therefore proves that the mechanism is via proteasomal degradation of the PD-L1 palmitoyltransferase DHHC3. Despite some effective vaccines and cancer screening for cervical cancer, around 600,000 new cases and 350,000 deaths, are reported annually worldwide. PD-1 inhibitor has achieved surprising clinical effects in increasing overall survival (OS) of patients with recurrent cervical cancer, compared to platinum-based chemotherapy [15]. It is widely realized that drug resistance is an important problem in patients with advanced stage cervical cancer. Therefore, we applied a combination treatment of the Peptide-PROTACs + Cisplatin on Hela and C33A cells, and measured a significant synergistic effect compared to cisplatin monotherapy (Figs. 1k and S12). Proliferative and apoptosis assays both demonstrated an additive effect of the Peptide-PROTACs in combination with cisplatin in cancer cells (Fig. S11). These results demonstrate that Peptide-PROTACs can inhibit cell proliferation and tumor growth by inducing cell apoptosis in both C33A and Hela cells. It has been demonstrated that the function of cell-intrinsic PD-1 in different cancer were different [11, 16]. Our results suggested blockage of the cell-intrinsic PD-1/PD-L1 maybe the reason why combined PD-1/PD-L1 inhibitors and cisplatin had an increased efficacy in cervical cancer cells.

**Fig. 1:**
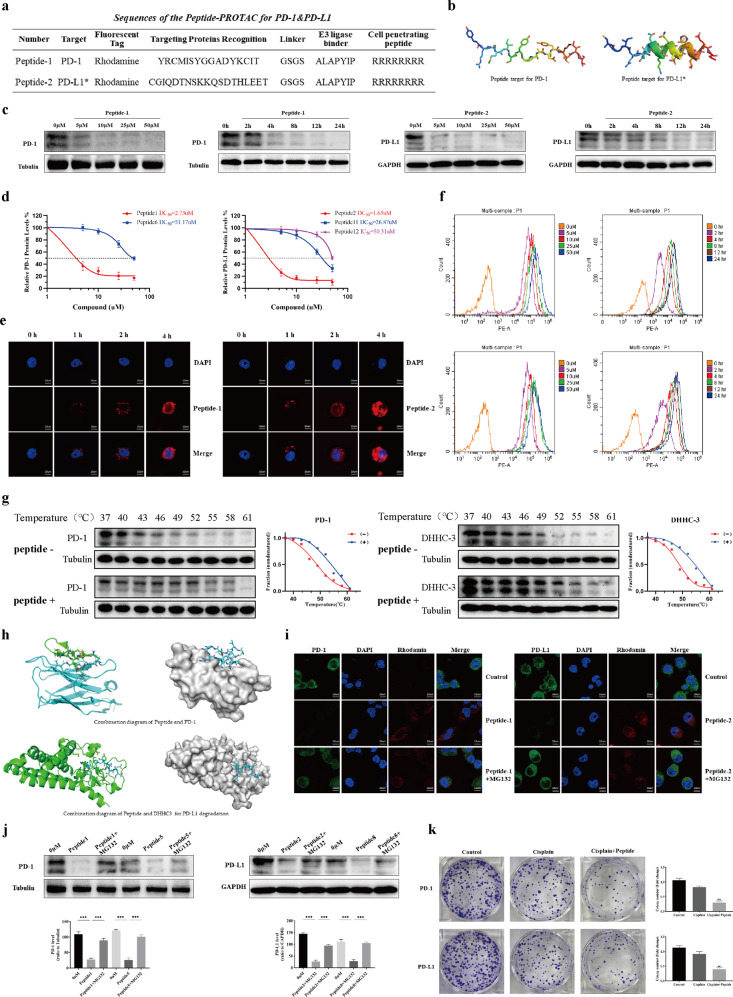
Peptide-PROTAC targeting of PD-1/PD-L1 in human cervical carcinoma. **a** The schematic illustrates the locations of the CPP sequence, TPR Peptide sequence, Peptide Linker, and ERP sequence. Peptide 1: the initial TPR sequence of Peptide 1 that target PD-1, and *Peptide 2 induced the decrease of PD-L1 dramatically by mean of the initial TPR sequence that target PD-L1 palmitoylation. **b** Schematic of peptides for PD-1 and PD-L1 palmitoylation. The AlphaFold 2 Artificial Intelligence system was used to generate three-dimensional structures of Peptide 1 (targeting PD-1) and Peptide 2 (targeting DHHC3, upstream of PD-L1) and visualizations were performed using the PyMOL software. **c** Western blotting analysis of C33A cells after treatment with the Peptide-PROTAC (Peptide 1 and Peptide 2) degraders at the indicated doses and timepoints. Values are presented as mean ± SEM. One-way ANOVA followed by Tukey’s post hoc test (*n* = 3): *P < 0.05, **P < 0.01, ***P < 0.001. **d** DC50 (50% protein degradation concentration) of Peptides targeting PD-1/PD-L1 in C33A cells. Both Peptide 1 and 6 target for PD-1, but Peptide 1 had a much better degradation effect than Peptide 6. Peptide 2 targeting for PD-L1 palmitoyltransferase ZDHHC3 (DHHC3) had a much better PD-L1 decrease effect than Peptide 11 targeting for PD-L1 directly and Peptide 12 which is the inhibition peptide without EPR sequence. **e** C33A cells were treated with 10 μM of rhodamine-labeled Peptide-PROTAC targeting PD-1 or PD-L1. After 4 h, the distribution of peptides in C33A cells was observed by confocal laser microscopy. Red; Peptide-PROTAC, blue; nucleus. Scale bar, 10 μM. **f** C33A cells were incubated with rhodamine-labeled Peptide-PROTAC targeting PD-1 or PD-L1 at the indicated concentrations and timepoints, then fluorescent cells were quantified by flow cytometry. Results are reported for 100,000 cells. *n* = 3. **g** CETSA-based determination of binding between Peptide (1 and 2) and PD-1/DHHC3. CETSA curves of PD-1/DHHC3 in C33A cells were determined in the absence and presence of 25 μM Peptide and analyzed by western blotting. GAPDH was used as an internal control. The band intensities of PD-1/DHHC3 were normalized with respect to the intensity at 40 °C. (−) without Peptide-PROTAC treatment, (+) with Peptide-PROTAC treatment. **h** Combination diagram of Peptide and PD-1/DHHC3. **i** Confocal microscopic images show the effects of Peptide-PROTAC (10 μM) and rescue by MG132 (4 mM) in C33A cells. Scale bars, 10 μM. **j** Western blotting analysis shows the PD-1 and PD-L1 levels after treating C33A cells with Peptide-PROTAC for 4 h. MG132 (4 mM) was added for 4 h before cell harvest. Values are presented as mean ± SEM. One-way ANOVA followed by Tukey’s post-hoc test (*n* = 3): ***P < 0.001. **k** C33A cells were treated with Peptide-PROTAC (10 μM) targeting either PD-1 or PD-L1 + cisplatin (15 μM). Colony formation assays were performed following treatment with cisplatin or cisplatin + Peptide-PROTAC treatments.

In summary, we have designed the first series of peptide-induced PROTACs that are able to induce cell-intrinsic degradation of the checkpoint protein PD-1 and its ligand PD-L1. Compared to the conventional ternary small molecule PROTACs, Peptide-PROTACs are easy to design, with an abundance of known PPIs that can be utilized, reducing the cost of ligand development for POIs. We demonstrated that degradation of PD-1 or PD-L1 via our novel Peptide-PROTACs induced cancer cell death, giving an exciting potential solution to the drug resistance seen with current clinical PD-1/PD-L1 inhibitors. Although further studies are required to test this system in vivo, the high-efficiency degradation at low Peptide-PROTAC doses will hopefully lead to better clinical effects than current PD-1/PD-L1 inhibitors in cancer patients.

## Supplementary information

Supplementary Material

Supplemental Material of original data of Western Blot

Reproducibility checklist

## Data Availability

The experimental data sets generated and/or analyzed during the current study are available from the corresponding author upon reasonable request. No applicable resources were generated during the current study.
