# Supplementary material for: High-potency PD-1/PD-L1 degradation induced by Peptide-PROTAC in human cancer cells
Source: Cell Death Dis. 2022 Nov 4;13(11):924. doi: 10.1038/s41419-022-05375-7 (PMC9636179; doi:10.1038/s41419-022-05375-7)
Supplement: Supplementary file 1 — Supplementary Material [file 41419_2022_5375_MOESM1_ESM.docx]

Supplementary Materials for

**High-potency Peptide-PROTAC degradation targeting for PD-1/PD-L1 pathway in human cancer cells**

1. **Biological and biochemical methods**

**Cell lines and cell culture**

The human C33A, Hela, Siha and MOLT-4 cell lines were obtained from ATCC. C33A cells were cultured in MEM with 20% FBS and 1% penicillin-streptomycin in a 37 °C incubator containing 5% CO2. Siha and Hela cell lines were cultured in DMEM with 10% FBS and 1% penicillin-streptomycin in a 37 °C incubator containing 5% CO2. MOLT-4 cells were cultured in RPMI-1640 with 10% FBS and 1% penicillin-streptomycin in a 37 °C incubator containing 5% CO2. The cells were treated with peptides for 4 h before proceeding with experiments.

**Reagents**

Primary antibodies: PD-L1 (Rabbit mAb, ab205921, 1:1000), GODZ/DHHC-3 (Rabbit mAb, ab31837, 1:800), and PD-1 (mouse mAb, ab89828, ab52587, 1:800) were purchased from Abcam. PD-1 (mouse mAb,TA806806,1:1000) were purchased from OriGene. Ki67 (Rabbit mAb, 27309-1-AP, 1:200), α-Tubulin (mouse mAb, 66031-1-Ig, 1:10000), and GAPDH (mouse mAb, 60004-1-Ig, 1: 10000) were purchased from Proteintech. Rabbit (SA00001-2, 1:10000)/mouse (SA00001-1, 1:10000) conjugated secondary antibodies were purchased from Proteintech. Cy3-conjugated goat anti-rabbit IgG(H+L) (GB21303, 1:500) was purchased from Servicebio. MG132 (IZL-3175-v) was purchased from Peptide International.

**Western blotting**

Following treatment, protein lysates were obtained from cultured cells, and western blotting was performed according to a standard protocol. Briefly, cervical cancer cell pellets were collected in EP tubes, lysed in RIPA buffer, and the protein concentration of cell lysates were measured by Bradford assay. Equal quantities of protein were separated by 10% SDS-PAGE and then transferred onto a nitrocellulose membrane. After blocking the membrane in 5% skim milk at room temperature, the membrane was incubated with primary antibody as follows: PD-L1 (MW: 40-60kDa,1:2000 dilution); PD-1 (MW: 35kDa, 1:1000 dilution); α-Tubulin (MW: 50kDa; 1:5000 dilution), and internal control GAPDH, prepared in 1% BSA in TBST at 4 °C overnight. Next, the membrane was washed and stained with secondary antibody for 1 h at room temperature. Signals were then detected using an ECL system (Vazyme, Jiangsu, China) and a ChemiDoc XRS imaging system (Bio-Rad Laboratories Inc., Heracles, CA, USA). Image J 2.0 software was used for semi-quantification.

**Cellular thermal shift assay (CETSA)**

Cells were collected into PCR-tubes, pelleted by centrifugation, and, after careful removal of the supernatant, resuspended in PBS. The tubes were kept at room temperature until the heat treatment step, which involved heating the PCR-tube strips to their designated temperature (nine temperature endpoints between 37 and 61 °C) for 3 min (Veriti thermal cycler, Applied Biosystems), then cooling for 3 min at room temperature. The cell pellet was lysed with RIPA buffer at 4 °C for 30 min and centrifuged at 20000 × g for 20 min at 4 °C. The detection and quantification of the soluble protein was then achieved by western blotting.

**Flow cytometry**

Peptides were labeled with rhodamine, and, following treatment, labeled cells were harvested and resuspended in PBS. The labeled cells were analyzed using flow cytometry (Beckman, cytoFlex S).

**Cell viability assay**

Cells were seeded into 96-well plates at a density of 4000 cells/well, cultured for 16–24 h, and five technical wells were replicated for each peptide treatment. Next, 20 μl of MTT solution (5 mg/ml) was added to each well, and cells were incubated for 4 h at 37 °C with 5% CO2. After removal of the reagent, 150 μl of DMSO was added to each well. The plate was shaken gently for 10 min, and OD absorbance values were measured at 490 nm using a 96-well plate reader. Cell viability was calculated using the following formula: cell viability (%) = (OD of control-OD of treatment)/(OD of control-OD of blank)×100. The assay was repeated 3 times. GraphPad Prism 5 software was used to assess differences between groups and plot histograms.

**Colony formation assay**

C33A and Hela cells were seeded into 6-well plates, incubated for 15 days, then fixed with 10% formalin for 30 min. After washing twice with PBS, the plates were stained with 0.5% crystal violet at room temperature. After washing twice with PBS, the plates were dried and the colonies were observed under a microscope.

**TUNEL Assay**

TUNEL assays were performed on cervical cancer cells using the one-step TUNEL apoptosis assay kit (Beyotime, Shanghai, China) according to the manufacturer’s instructions. After treatment, samples were incubated with TUNEL reaction mixture for 1 h at 37 °C in the dark and then washed twice in PBS. The condensed or fragmented nuclei of apoptotic cells were observed using fluorescence microscopy (Olympus IX73, Japan) at 200× magnification.

**Immunofluorescence staining of Ki67**

The cell slides were washed twice with PBS, then fixed in 4% formaldehyde for 30 min, and washed with PBS. Cells were permeabilized with 0.3% Triton X-100 for 10 min, then blocked with BSA for 10 min at 37 °C, and incubated with anti-KI67 antibody (1:100) overnight at 37 °C in a wet-box. After washing twice with PBS, blastocysts were incubated with Cy3-conjugated goat anti-mouse antibody and counterstained with DAPI to visualize nuclei. The slides were sealed with anti-fluorescence quenching tablets. The fluorescent signals were examined under a fluorescence microscope (Olympus IX73, Japan).

**Immunofluorescence microscopy**

To observe the distribution of peptides and their interactions with the targeted POIs, we labeled peptides with rhodamine, and located the targeted proteins by immunofluorescence. All experimental procedures were performed in the dark. Cells were fixed on coverslips in 4% paraformaldehyde for 15 min at room temperature and permeabilized for 15 min with 0.3% Triton-X100. After blocking with 1% bovine serum albumin (BSA) for 30 min, cells were incubated for 2 h at room temperature with primary antibody against PD-L1 (1:500) or PD-1 (5 μg/ml) diluted in PBS with 1% BSA. Following several rinses with PBS, cells were incubated for 1 h at room temperature with secondary antibodies in 1% BSA/PBS and counterstained with DAPI to observe the nuclei. The slides were sealed with anti-fluorescence quenching tablets. Cells were viewed and imaged using Nikon Eclipse Ni-U microscope (Nikon, Japan) equipped with ProgRes MFcool (Jenoptik AG, Germany), a high-definition camera.

**T cell function assay by a model of co-culture of cancer cells and T cells**

Establishment of co-culture of human cervical cancer cells with T cells: Firstly, peripheral blood mononuclear cells (PBMCs) were isolated by density gradient centrifugation from the whole blood, diluted with PBS, and the CD3+ cells were separated with the Easy Sep Human T cell Isolation Kit (STEMCELL Technologies Inc., Vancouver, Canada) and then resuspended in PBS prior to incubation. The cervical cancer cells (C33A) were resuspended with culture medium after 10ug/ml of mitomycin treatment and added into 96-well ULA round-bottomed microplate mixing with T cells at a ratio of 1:2, then centrifuged (350g×5min). IFN-γ in the culture supernatants was quantified with the Enzyme-linked immunosorbent assay, ELISA kit (Nebioscience, China) after the increasing doses of Peptide-PROTAC treatment. All the experiments were repeated three times.

**Statistical analysis**

All experiments were performed at least 3 times. Quantitative data are expressed as the mean ± standard deviation. Statistical analyses were performed by GraphPad Prism 5 and SPSS17.0 software. The Student’s t test was used to compare the differences between the treated groups and the corresponding control groups. p < 0.05 was considered statistically significant.

**2. Computer-aided drug design (CADD)**

**Peptide design and interaction with PD-1/PD-L1**

The sequence of Peptide 1 was taken from Reference 12, whereas Peptide 2 was designed using the AlphaFold 2 AI system to generate the 3D structures and amino acid sequences (targeting DHHC3), using pdb70 as the template. Interactions between peptides and POIs were assessed by separately aligning the peptide sequences with the POIs based on the crystal structures. Energy minimization was performed by Molecular Operating Environment (MOE) software, and visualizations were performed using PyMOL software.

**Molecular docking for DHHC3-targeting peptides**

The DHHC3 structure was predicted using the Alphafold 2 and Robetta systems, and then homology modeling and AI drug virtual screening were implemented using SWISS-Mode server homology modeling. The HPEPDOCK docking server was used to carry out the molecular docking simulation. The base peptide sequences of the shift window were from Reference 5, and we further optimized the sequences by molecular docking. With the modeled DHHC3 structure as a receptor and the peptide sequence in FASTA format as an input, we performed the docking simulation with default parameters and further analyzed 10 predicted conformers (poses). The highest scoring peptide sequence alignment was chosen as the potential POI ligand and taken forward for synthesis of the Peptide-PROTAC. Visualizations were performed using PyMOL software.

**Virtual screening of PD-L1 palmitoylation (DHHC3) and designed peptides**

**3. Mass spectrum analysis part**

**Parallel reaction monitoring (PRM) assay**


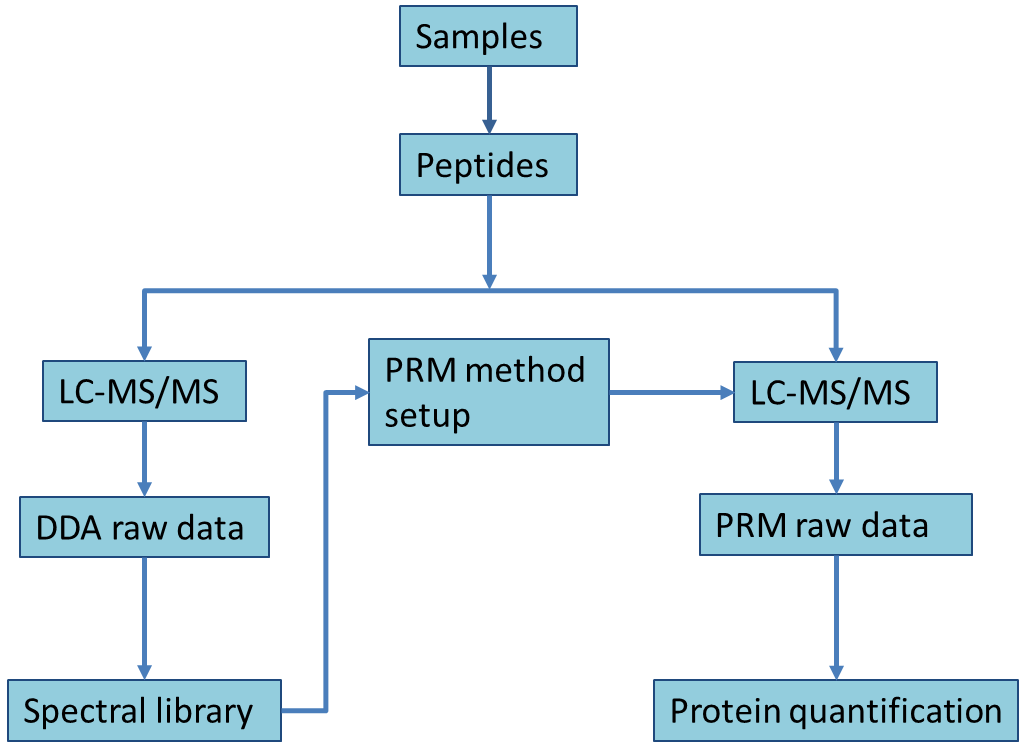


Technique procedure of PRM

**Sample preparation**

Added the responsive reagents (1%SDC/100 mM Tris-HCL pH=8.5/10 mM TCEP/40 mM CAA) into the samples and incubated at 60℃ for 1 hour. One-step protein denaturation, reduction and alkylation: Diluted by Millipore ultrapure water and added 0.25% Tripsin (1:50 vs proteins), incubated at 37 ℃ for overnight for enzymatic cleavage. Then added TEA to terminate the enzyme reaction. After centrifugation (16,000 ×g), the supernatant was subjected to peptide purification using SDB desalting column. After the solution was drained, samples were stored frozen at −20°C.

**Mass spectrum process**

Thermo Scientific™ Orbitrap Exploris™ 480 mass spectrometer was used. Prior to analysis, peptides solution was added by the autosampler and bound to a C18 guard column, then peptides were eluted to an analytical column (75 μm×250 mm, 3 μm particle size, 100 Å pore size, Acclaim PepMap C18 column, Thermo) and separated. Two mobile phases (mobile phase A: 0.1% formic acid and mobile phase B: 80% ACN, 0.1% formic acid) were used to establish a 60-minutes gradient. Flow rate was at 300 nl/min. For PRM analysis, each scan cycle consisting of one full-scan mass spectrum (R = 60 K, AGC = 3e6, max IT = 50 ms, scan range = 300–1200 m/z) and several PRM MS2 scans (R = 15 K, AGC = 2e5, max IT = 50 ms) for the specific peptides were followed. The HCD collision energy was set to 28. The screening window for the quadrupole is set to 1.6 Da.

**Data analysis**

PRM method establishment and protein quantification were performed using Skyline software to obtain quantitative information of target proteins and peptides. The quantitative value of the protein comes from multiple peptide precursor ions (precursor), and the quantitative information of the precursor comes from the accumulation of the integrated peak areas of 3 to 5 product ions. The obtained protein quantification value was normalized by the total TIC signal between different samples and used for subsequent quantitative comparison.

1. **Supplementary data**

| **Number** | **Target** | **Fluorescent Tag** | **Targeting Proteins Recognition** | **Linker** | **E3 ligase binder** | **Cell penetrating peptide** |
| --- | --- | --- | --- | --- | --- | --- |
| Peptide-3 | PD-1 | Rhodamine | YRCMISYGGADYKCIT | GSGS | RRRG | RRRRRRRR |
| Peptide-4 | PD-1 | Rhodamine | YRCMISYGGADYKCIT | GSGS | AVPF | RRRRRRRR |
| Peptide-5 | PD-1 | Rhodamine | YRCMISYGGADYKCIT | - | RRRG | YGRKKRRQRRR |
| Peptide-6 | PD-1 | Rhodamine | FNWDYSLEELREKAKYK | - | RRRG | YGRKKRRQRRR |
| Peptide-7 | PD-L1^*^ | Rhodamine | CGIQDTNSKKQSDTHLEET | GSGS | ALAPYIP | YGRKKRRQRRR |
| Peptide-8 | PD-L1^*^ | Rhodamine | CGIQDTNSKKQSDTHLEET | GSGS | RRRG | RRRRRRRR |
| Peptide-9 | PD-L1^*^ | Rhodamine | CGIQDTNSKKQSDTHLEET | GSGS | AVPF | RRRRRRRR |
| Peptide-10 | PD-L1^*^ | Rhodamine | CGIQDTNSKKQSDTHLEET | - | RRRG | YGRKKRRQRRR |
| Peptide-11 | PD-L1 | Rhodamine | MPIFLDHILNKFWILHYA | - | RRRG | YGRKKRRQRRR |
| Peptide-12 | PD-L1  (Peptide Inhibitor) | Rhodamine | CGIQDTNSKKQSDTHLEET | - | - | YGRKKRRQRRR |

**
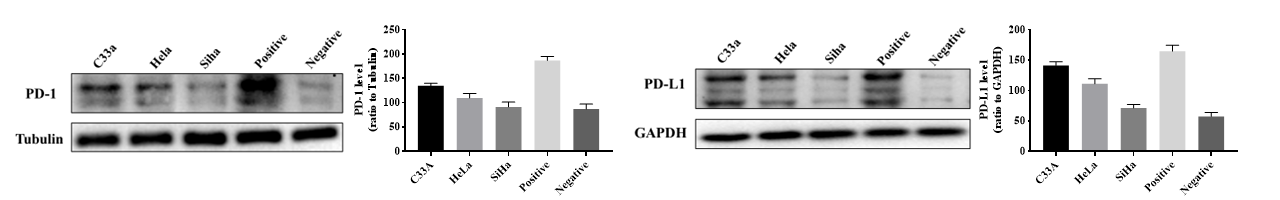
Supplementary information, Table S1. Sequences of the Synthesized Peptide- PROTACs.** (*Peptide induced the decrease of PD-L1 by mean of the initial TPR sequence that target PD-L1 palmitoylation.)

**Supplementary information, Figure S1.** Expression of PD-1 and PD-L1 proteins in human cervical carcinoma cell lines. Expression of PD-1 and PD-L1 is high in C33A and HeLa cells, but low in SiHa cells. For PD-1, the negative and positive controls were untreated MOLT-4 cells, and MOLT-4 cells treated with 10 ng/ml Phorbol-12- Myristate-13-Acetate (PMA) +500 ng/ml lonomycin for 24 h, respectively. For PD-L1, the negative and positive controls were the A549 and MDA-MB-231 cell lines, respectively.


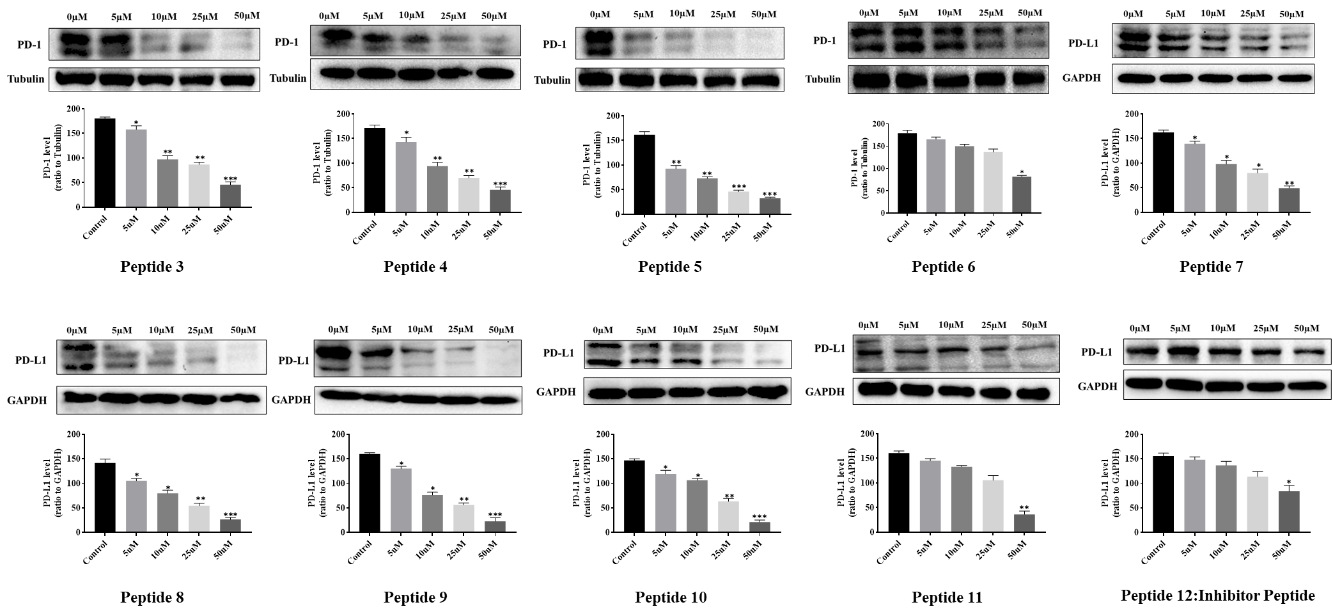
**Supplementary information, Figure S2.** Western blotting analysis shows the degradation levels of PD-1/PD-L1 in the C33A cell line after the indicated Peptide-PROTAC (Peptide 3-11) and Inhibitor Peptide (Peptide 12) treatments for 4 h, respectively. Values are presented as mean ± SEM. One-way ANOVA followed by Tukey’s post-hoc test (n = 3): *P < 0.05, **P < 0.01, ***P < 0.001.


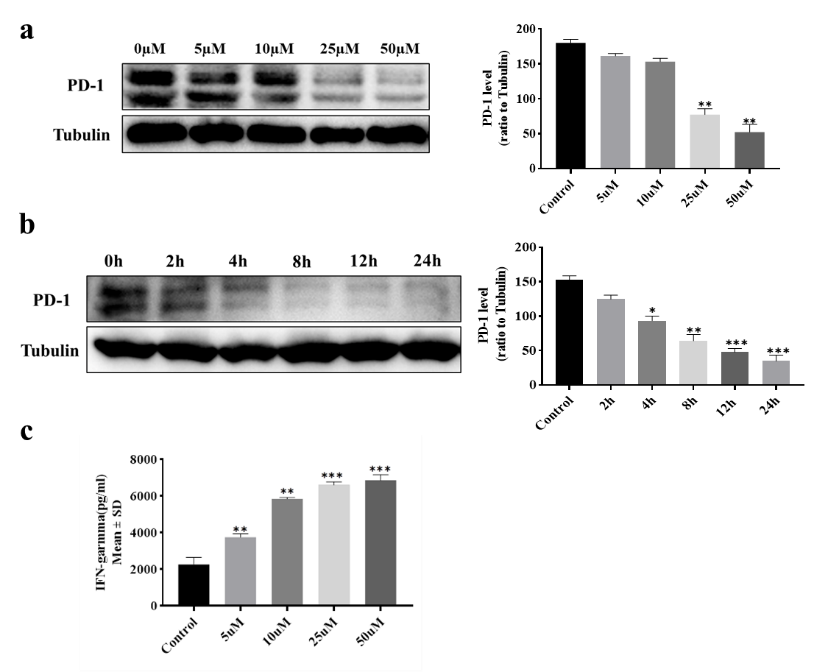
**
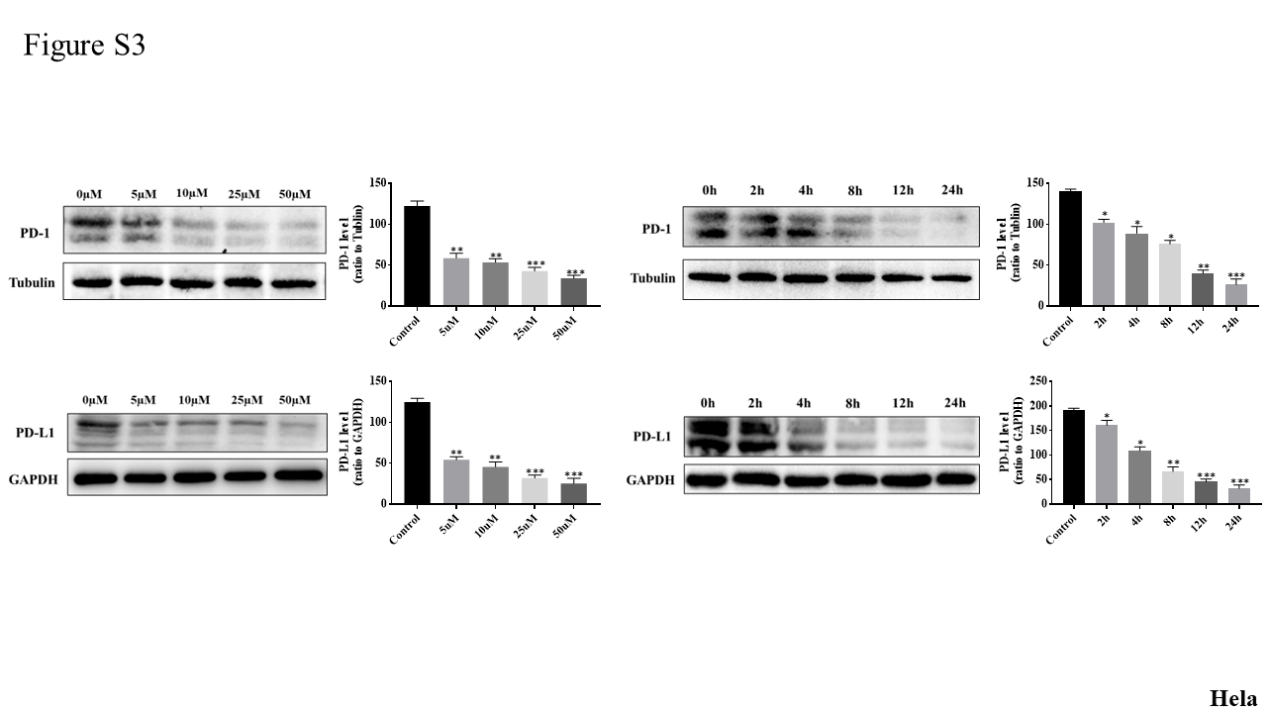
Supplementary information, Figure S3.** Western blotting analysis shows the degradation levels of Peptide 1 and Peptide2 targeting for PD-1 and PD-L1 respectively, in the HeLa cell line after the indicated Peptide-PROTACs treatments for 4 h. Values are presented as mean ± SEM. One-way ANOVA followed by Tukey’s post-hoc test (n = 3): *P < 0.05, **P < 0.01, ***P < 0.001.

**Supplementary information, Figure S4** Western blotting analysis of MOLT-4 cells after treatment with the Peptide-PROTAC (Peptide 1) degraders at the indicated doses (a) and timepoints (b). (c) Effects of Peptide1 on IFN-γin, C33A, and CD3^+^ T cell co-culture model. Values are presented as mean ± SEM. One-way ANOVA followed by Tukey’s post-hoc test (n = 3): *P < 0.05, **P < 0.01, ***P < 0.001.


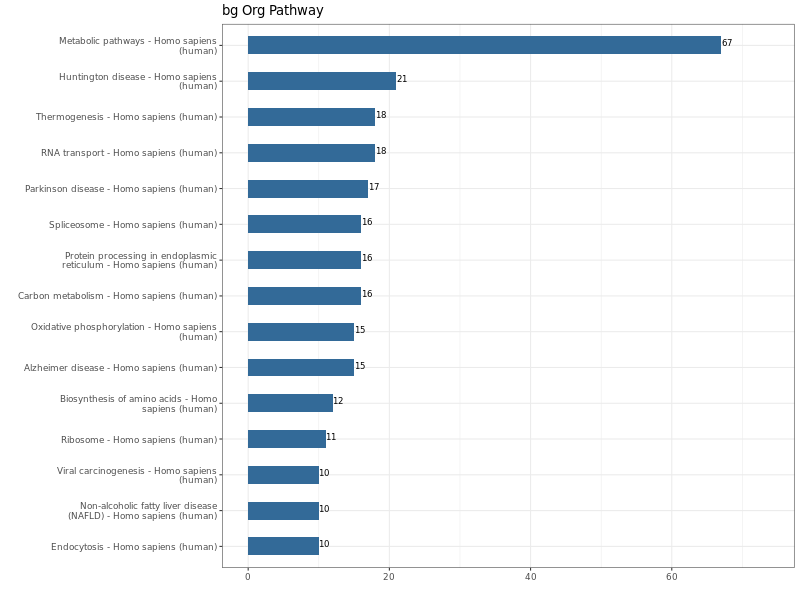
**Supplementary information, Figure S5.** Parallel Reaction Monitoring-Mass Spectrometry (PRM-MS) analysis: After determining the mass-to-charge ratio and retention time, the expression of the target peptides was measured. The quantification of peptides for PRM validation were performed using Skyline software which provided the necessary data for the POIs and peptides.

**a**

**b**


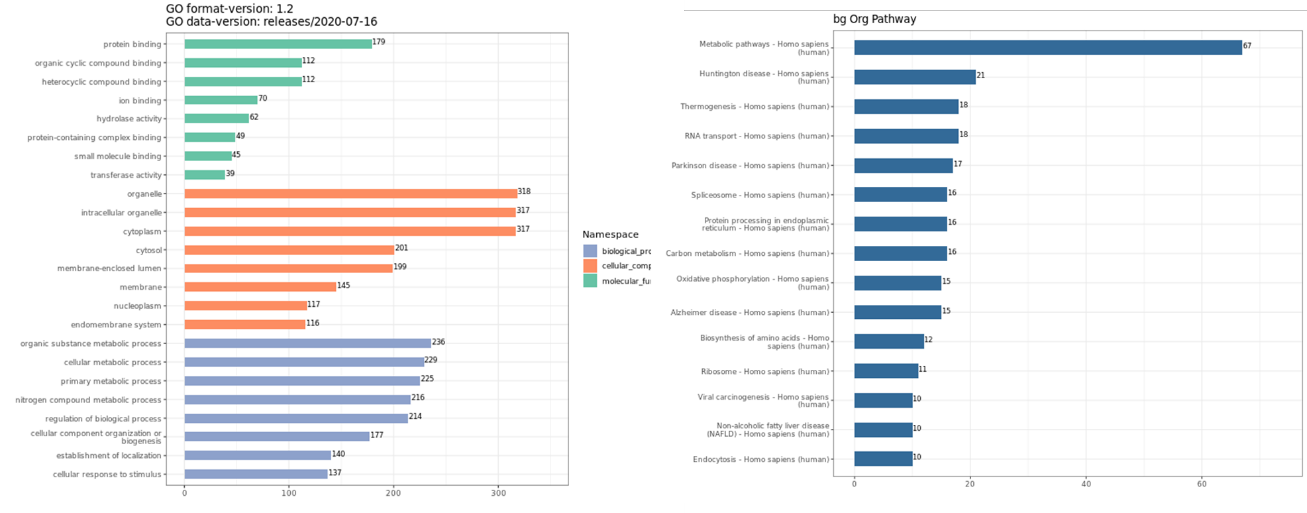


**a**

**b**

**Supplementary information, Figure S6.** **a** GO protein annotation data for peptides with a similar molecular weight as the targeting peptides, using the background protein as reference. **b** KEGG protein annotation data for peptides with a similar molecular weight as the targeting peptides, using the background protein as reference.


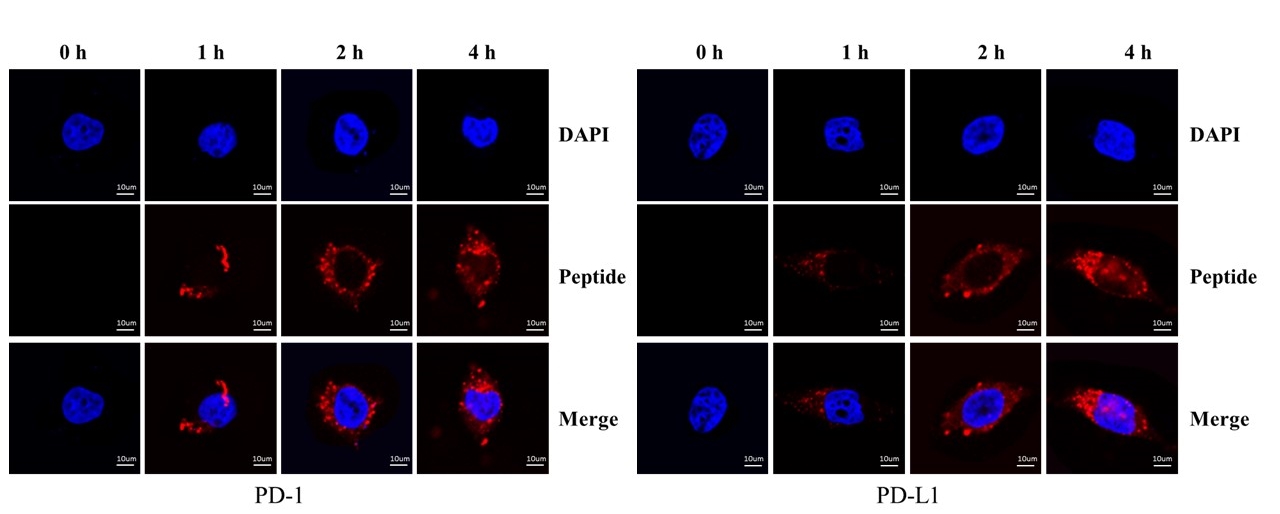
**
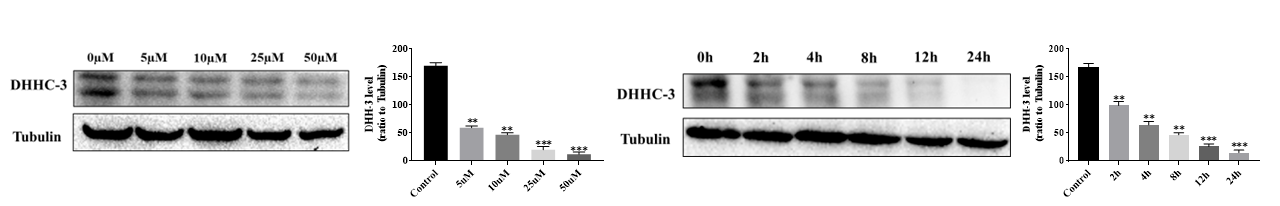
Supplementary information, Figure S7.** Western blotting analysis shows the degradation levels of DHHC3 in the C33A cell line after the indicated Peptide 2 treatments for 4 h. Values are presented as mean ± SEM. One-way ANOVA followed by Tukey’s post-hoc test (n = 3): **P < 0.01, ***P < 0.001.


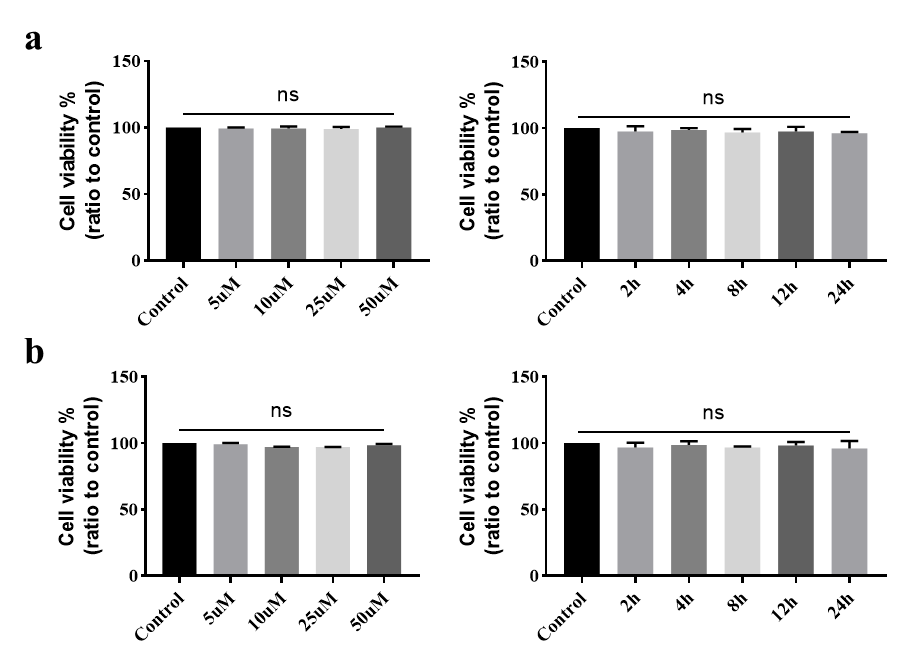
**Supplementary information, Figure S8.** HeLa cells were treated with Peptide-PROTAC (10 μM) targeting PD-1 or PD-L1 labeled with rhodamine. After 4 h, the distribution of peptides was observed by confocal laser microscopy. Red; Peptide-PROTAC, blue; nucleus. Scale bar, 10 μM.

**Supplementary information, Figure S9.** C33A cells (a) and Hela cells (b) were treated with Peptide-PROTAC targeting PD-1 or PD-L1 at the indicated concentrations and timepoints, and subsequently assessed by MTT assay. n = 3


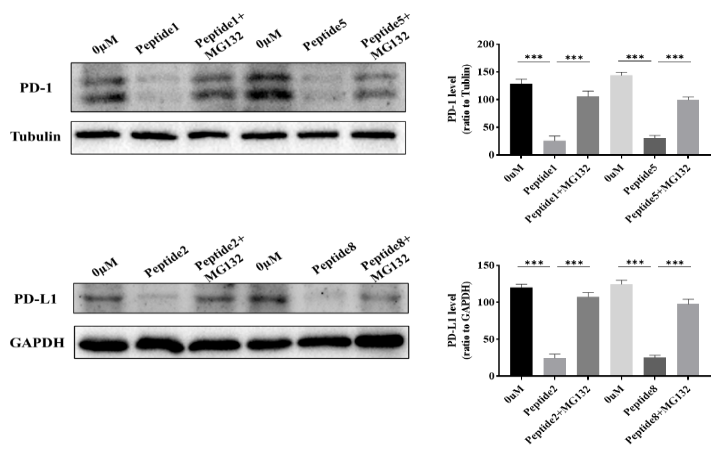
**Supplementary information, Figure S10.** Western blotting analysis showing protein levels of PD-1 and PD-L1 in HeLa cells following treatment with Peptide-PROTAC for 4 h. MG132 (4 mM) was added for 4 h before cell harvest. Values are presented as mean ± SEM. One-way ANOVA followed by Tukey’s post-hoc test (n = 3): ***P < 0.001.


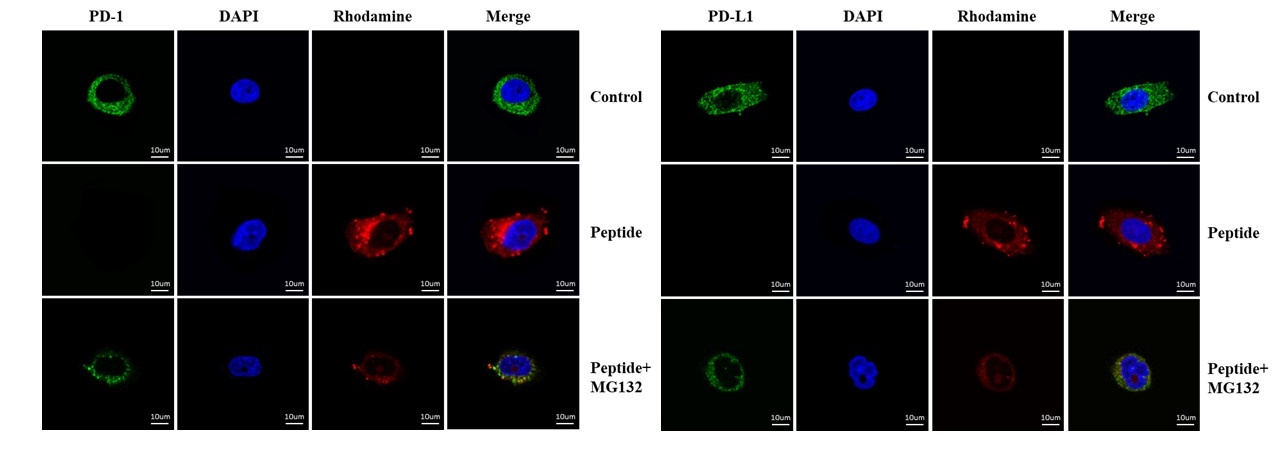
**Supplementary information, Figure S11.** Confocal microscopic images show the effects of Peptide-PROTAC (10 μM) and rescue by MG132 (4 μM) in HeLa cells. Scale bars, 10 μM.

**S****upplementary information, Figure S12.** C33A cells were treated with Peptide-PROTAC (10 μM) targeting either PD-1 (**a**) or PD-L1 (**b**) + cisplatin (15 μM) for 4 h and observed by immunofluorescence microscopy. PD-1 (c) or PD-L1 (d) + cisplatin (15 μM) for 4 h and observed by immunofluorescence microscopy. Ki67 staining (pink) was performed to observe cell proliferation. Proliferative index (%) = Proliferative cells/ total cells × 100. TUNEL staining (green) was performed to observe cell apoptosis. Apoptotic index (%) = Apoptotic cells/ total cells × 100. Scale bars, 100 μM. e Decreased survival of HeLa cell lines following treatment with cisplatin or cisplatin + Peptide-PROTAC. Colony formation assays were performed following treatment with cisplatin or cisplatin + Peptide-PROTAC treatments.
