# Supplementary figures and images for: High-potency PD-1/PD-L1 degradation induced by Peptide-PROTAC in human cancer cells
Source: Cell Death Dis. 2022 Nov 4;13(11):924. doi: 10.1038/s41419-022-05375-7 (PMC9636179; doi:10.1038/s41419-022-05375-7)

**Figure 1c**

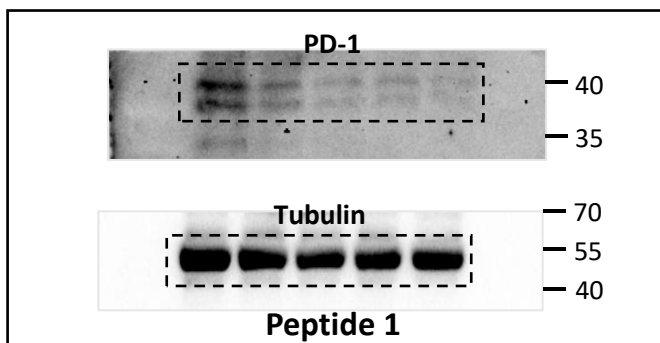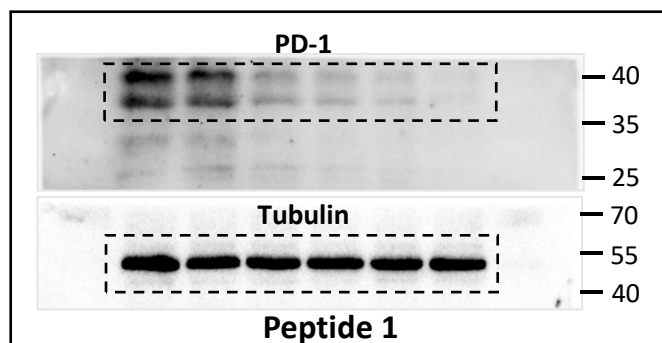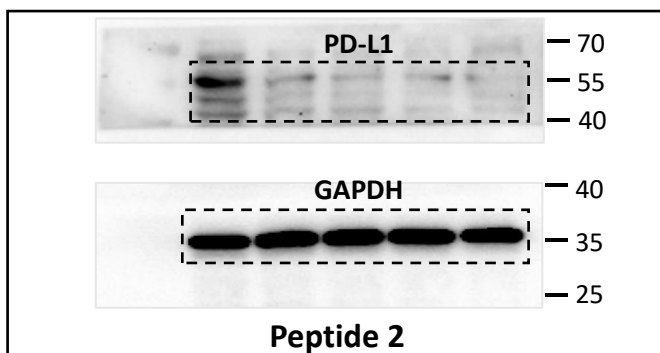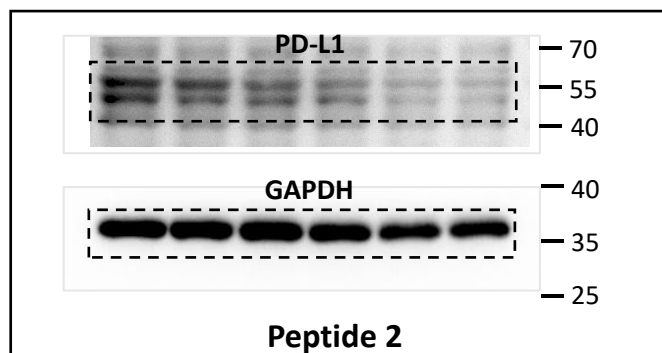

**Figure 1g**

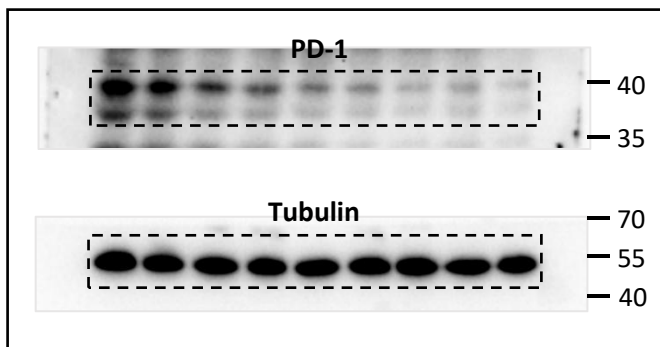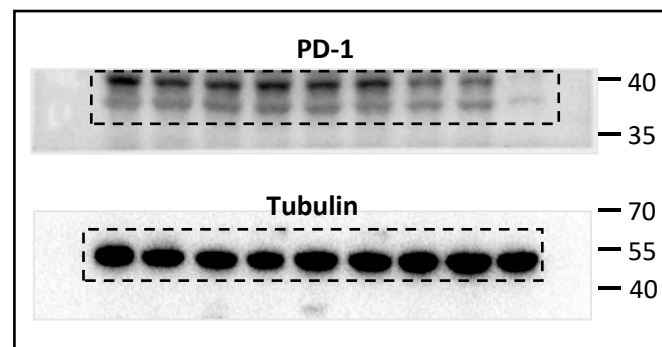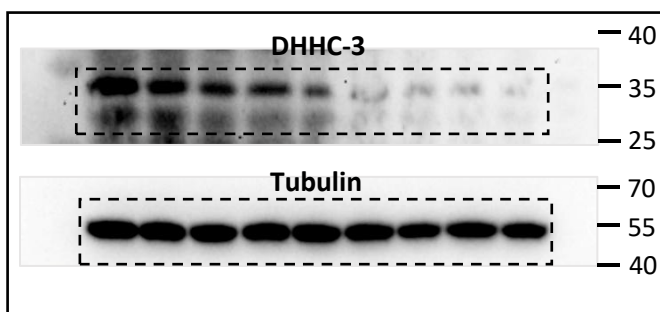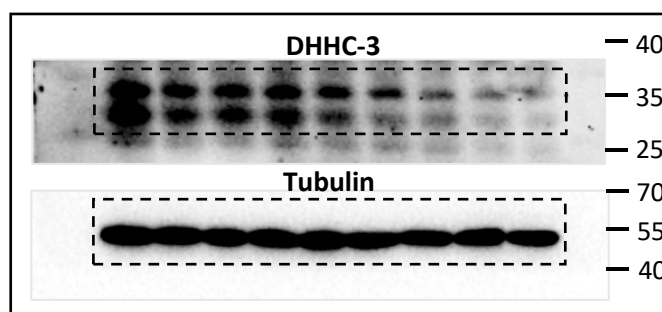

**Figure 1j**

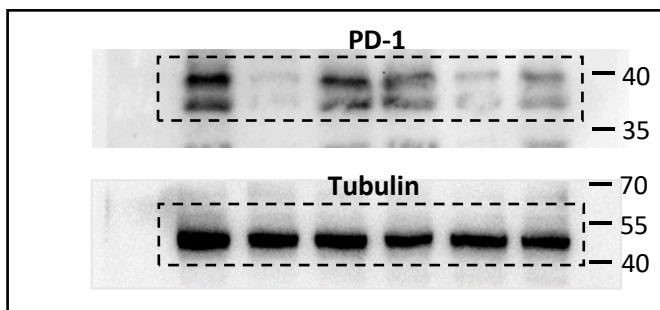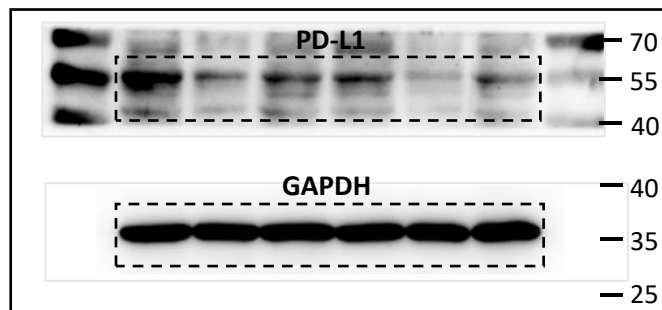

**Figure S1**

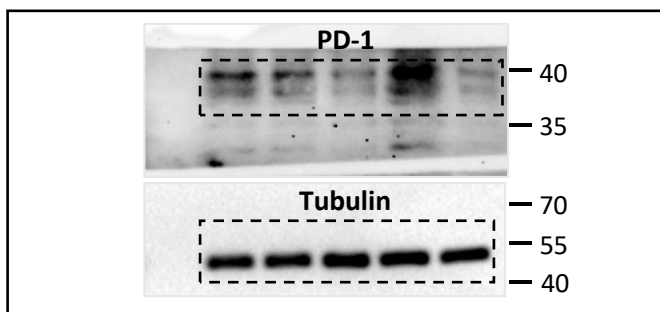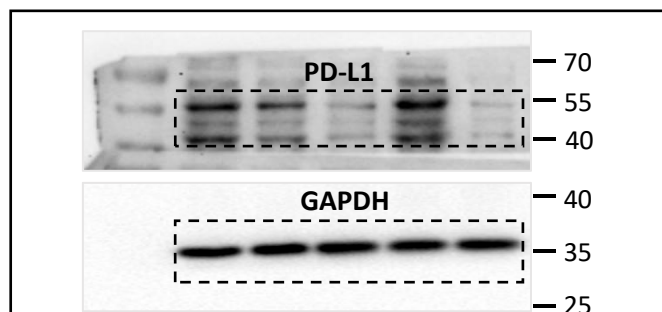

**Figure S2**

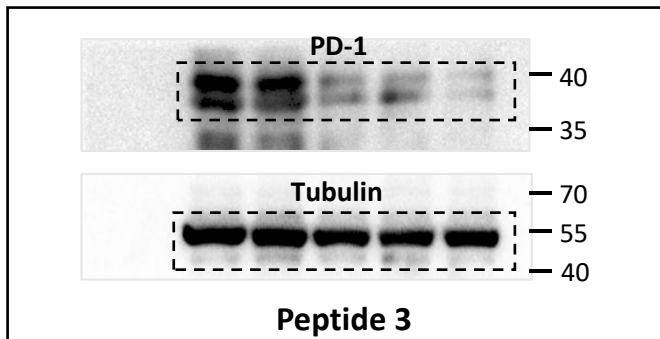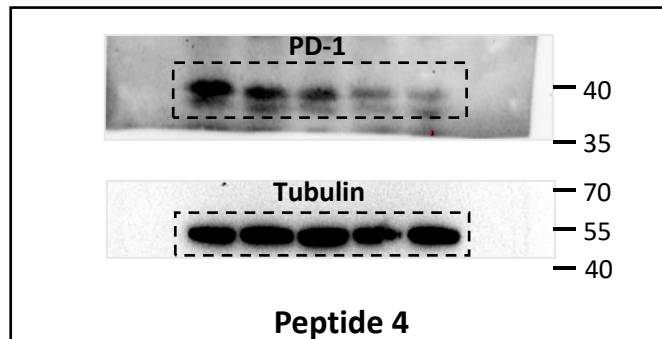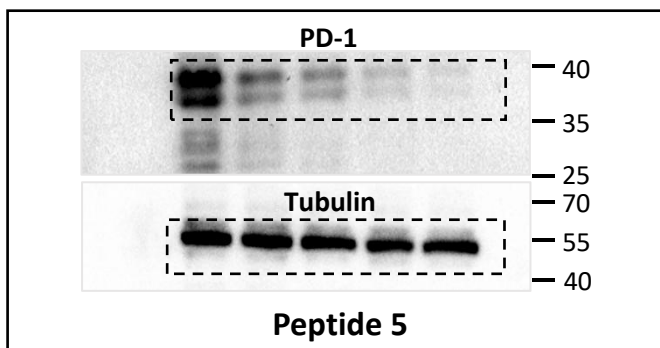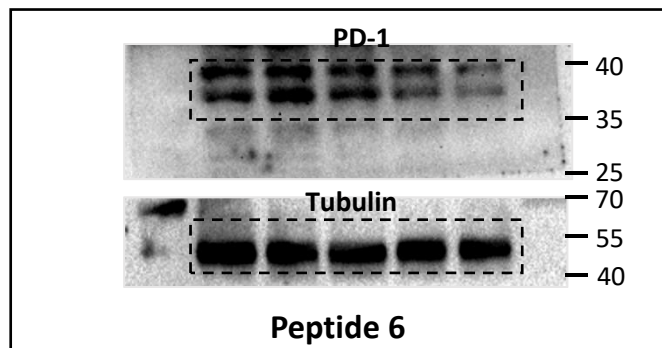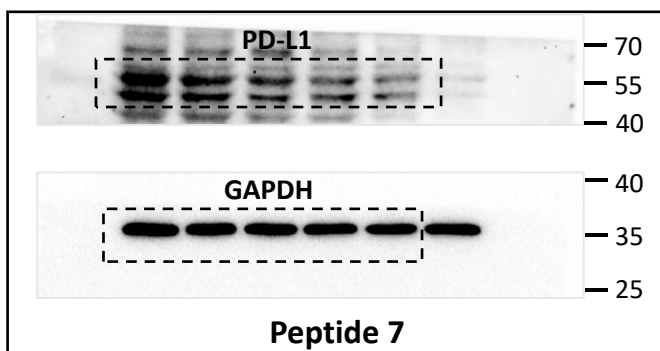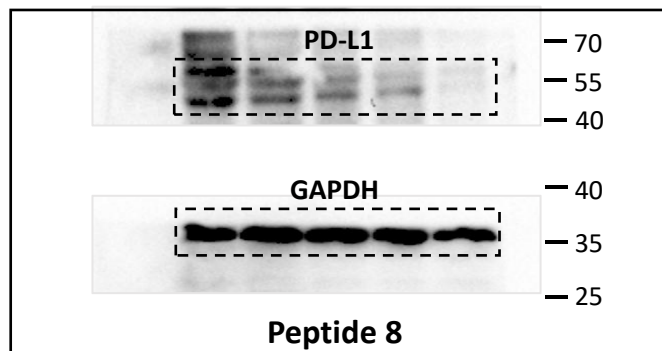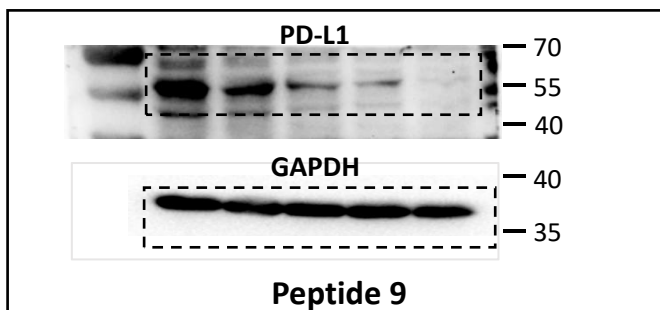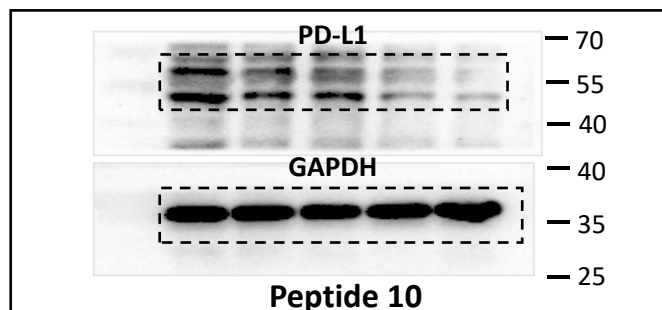

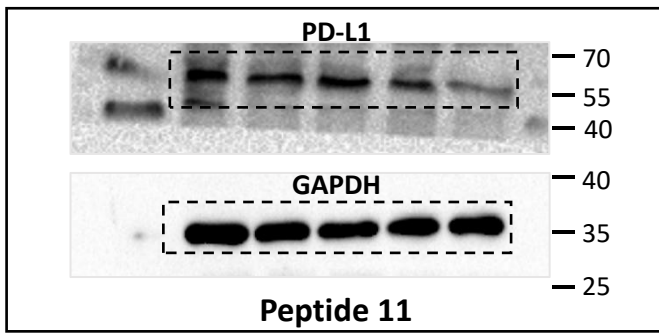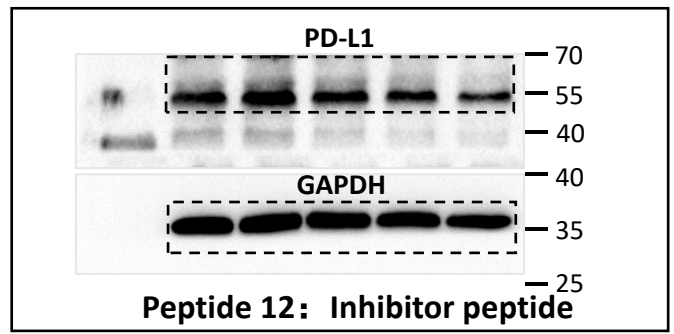

**Figure S3**

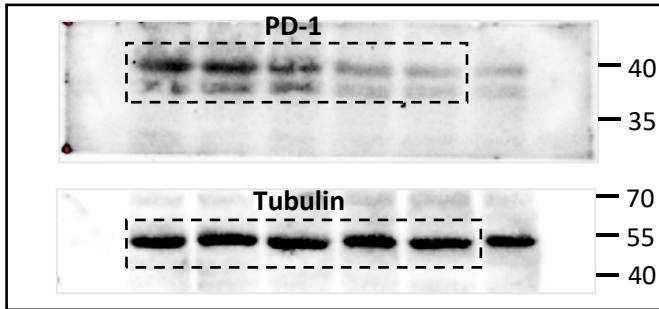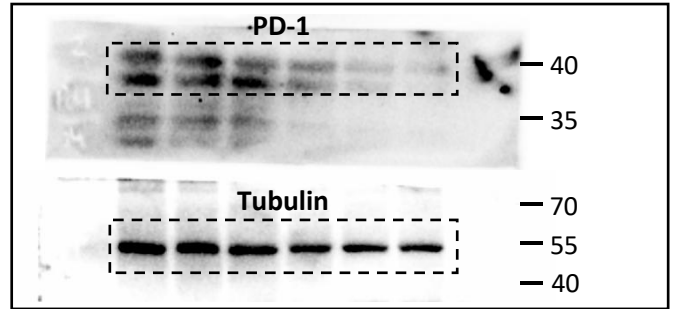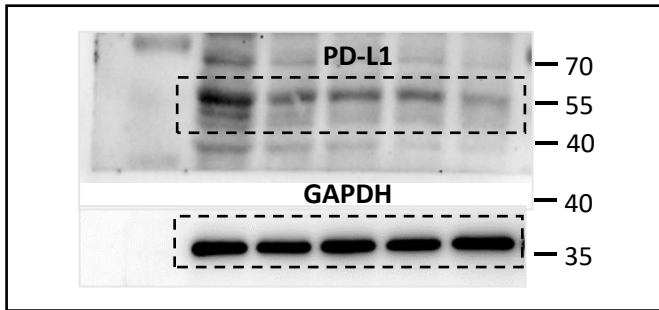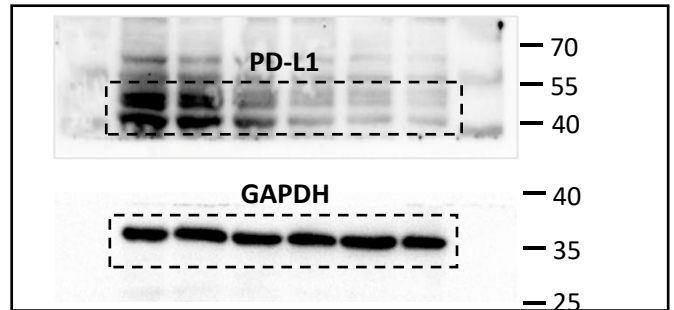

**Figure S4**

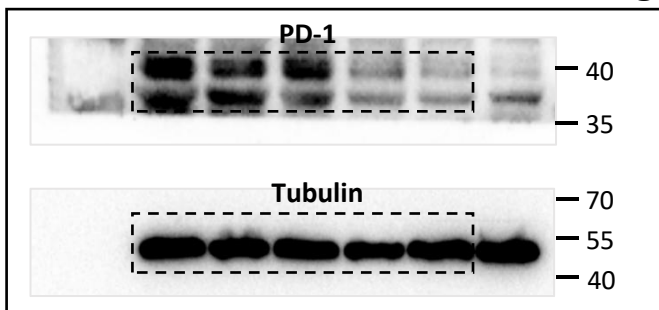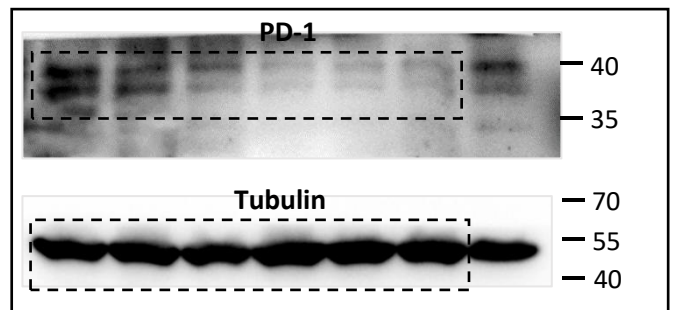

**Figure S7**

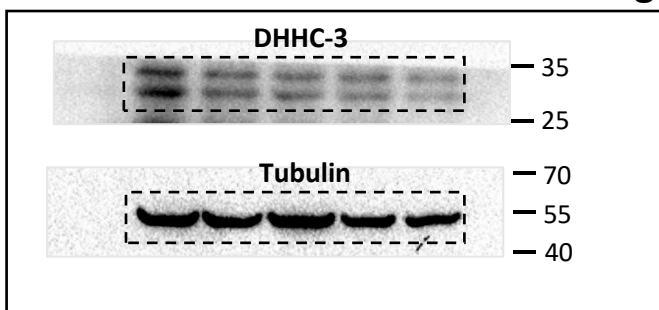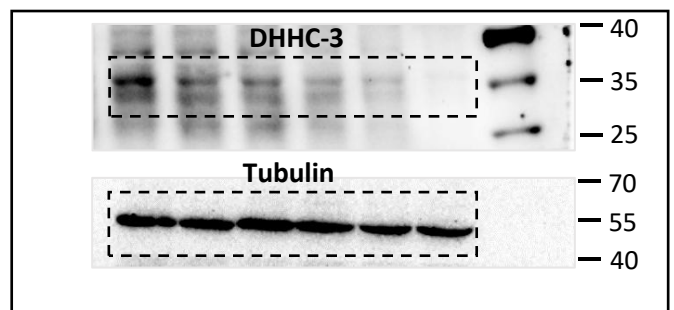

**Figure S10**

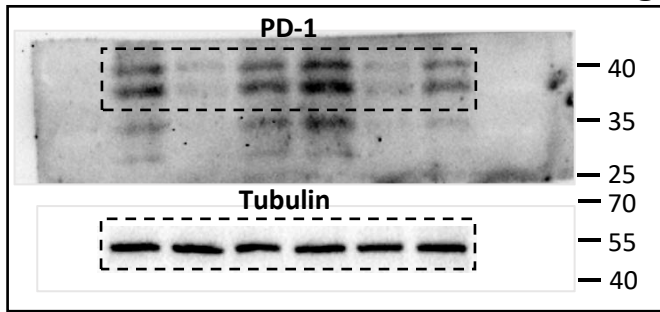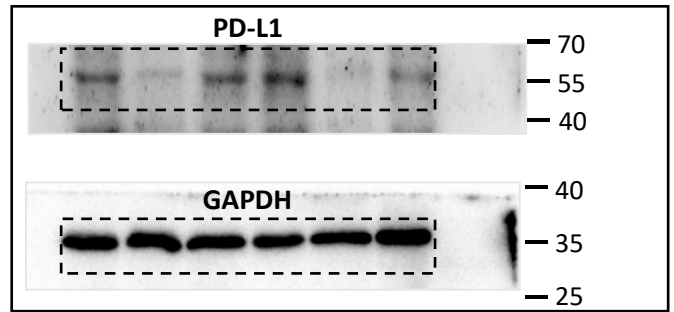

Supplement: Supplementary file 2 — Supplemental Material of original data of Western Blot [file 41419_2022_5375_MOESM2_ESM.pdf]
